# Multiple Simultaneous Mature Teratomas of the Spinal Cord in an Adult

**DOI:** 10.7759/cureus.10409

**Published:** 2020-09-12

**Authors:** Michelle DeWitt, Timothy E Richardson, Gaddum D Reddy

**Affiliations:** 1 Neurosurgery, State University of New York Upstate, Syracuse, USA; 2 Pathology, State University of New York Upstate, Syracuse, USA

**Keywords:** spinal teratoma, mature teratoma, multiple teratoma, adult teratoma

## Abstract

Teratomas of the spinal cord are rare tumors, particularly in adults, but there is an increasing body of literature documenting both their diagnosis and successful treatment with surgical resection. However, to date, the literature has largely characterized spinal teratomas as single solitary lesions. Here, we report on an adult patient who presented with signs of progressive lower extremity weakness. Imaging demonstrated two simultaneous lesions of the spine, an extramedullary lesion in the upper thoracic region and an intramedullary lesion in the mid-thoracic region. Both lesions were resected and pathologically determined to be mature spinal teratomas. To our knowledge, this is only the second report of this tumor presenting simultaneously at more than one location in the spine and the first time it has presented both as an intra-axial and extra-axial lesion. Our results suggest that the presence of more than one simultaneous lesion does not necessarily increase the risk of a more aggressive immature pathology.

## Introduction

Teratomas of the spine are rare. Even in the pediatric population, where teratomas as a whole are reported to account for approximately 3% of all tumors, spinal teratomas are infrequently seen [1]. In the adult population, this is even more infrequent, and recent comprehensive reviews of the literature have documented fewer than 150 reported cases in adults [2]. However, all but one of these reported cases presented as a single solitary lesion [3]. Similarly, the presence of an immature spinal teratoma in the adult is rare, with only two cases being reported in the literature [4,5]. With the relatively low numbers of each of these cases, it is unclear whether the presence of more than one lesion increases the likelihood of a more aggressive immature pathology. Here we present a case of a 73-year-old male who presented with progressive weakness of the bilateral lower extremities and was found to have both an intramedullary and an extramedullary spinal lesion. After simultaneous complete resections, both lesions were pathologically determined to be mature teratomas. 

## Case presentation

**Figure 1:**
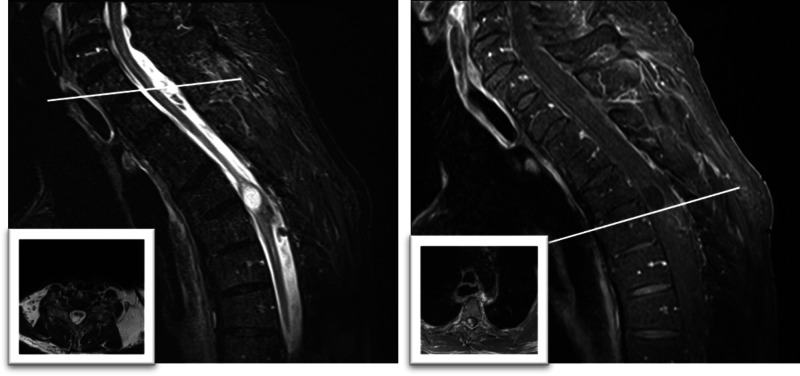
Preoperative Imaging Left: T2-weighted cervicothoracic MRI demonstrating an extra-axial lesion at the upper thoracic region and an intra-axial lesion in the midthoracic region. Inset: axial slice at the indicated level (C7-T1) demonstrating the extra-axial location. Right: T1-weighted cervicothoracic MRI with contrast of the same sagittal section as the image on the left, demonstrating heterogeneous contrast enhancement in the caudal lesion. Inset: Axial slice at the indicated level (T4-5) demonstrating the intra-axial location.

A 73-year-old male with an extensive medical history including dementia, prior esophageal carcinoma, and diabetes with associated neuropathy presented with complaints of progressively worsening weakness of the legs over several months. The family also endorsed multiple episodes of bowel and bladder incontinence over the past three days. Physical examination revealed good strength in the upper extremities, with only trace movement in the right lower extremity and no movement in the left lower extremity. He had a T4 sensory level and poor rectal tone. An MRI of the entire spine was performed and showed a 0.8 × 0.8 × 1.5 cm non-enhancing cystic extramedullary lesion at T1 as well as a 0.8 × 1.0 × 1.4 cm heterogeneously enhancing cystic intramedullary lesion at T4-5 (Figure 1). A subsequent MRI of the brain and metastatic workup were unremarkable. He was started on steroids and regained some strength in his right leg, but remained hemiplegic on the left. After a discussion of potential treatment options, the family and patient elected to undergo surgical resection for both diagnosis and treatment. 

Surgery

**Figure 2:**
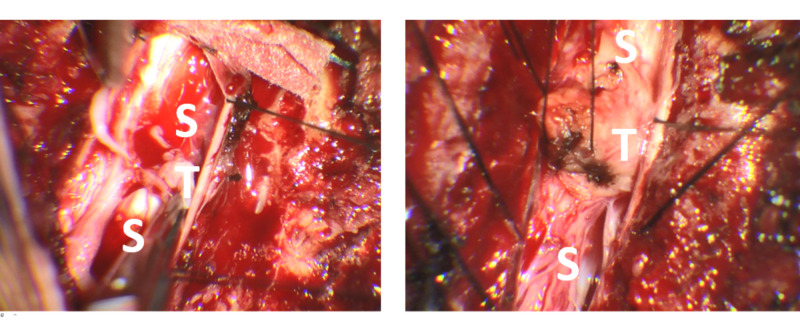
Intraoperative Images Left: Cystic tumor located in the extramedullary space as it is being dissected off the spinal cord after drainage of the cyst. Right: Intramedullary tumor with locally exophytic component. S: spinal cord; T: teratoma.

The patient underwent a C7-T1 and T4-6 laminectomy with complete microsurgical resection of both lesions. The rostral lesion was resected and noted to be completely extra-axial with only minimal adhesions to the spinal cord itself (Figure 2, left). The caudal lesion was also resected and noted to be primarily intramedullary with an exophytic component that extended into the intradural/extramedullary space (Figure 2, right). Frozen section diagnosis was consistent with teratoma, and no immature or malignant features were identifiable. The surgery was performed with both somatosensory evoked potential and motor evoked potential monitoring; however, there were poor signals at baseline, which were further diminished after resection of the larger lesion. 

Hospital course

After the surgery, the patient remained weak in both legs with a notable loss of strength in the right leg when compared to the preoperative exam. However, over the course of one week, he was able to regain some proximal motor function in both legs. Unfortunately, he also developed a postoperative ileus that after an extensive GI workup was felt to be secondary to Ogilvie’s syndrome and was placed on total parenteral nutrition (TPN) while being decompressed via nasogastric tube. Despite gradual improvements in his abdominal distention, the family requested removal of the TPN and palliative care measures only approximately two weeks after his surgery. He was subsequently discharged home with hospice care measures. 

Pathology

**Figure 3:**
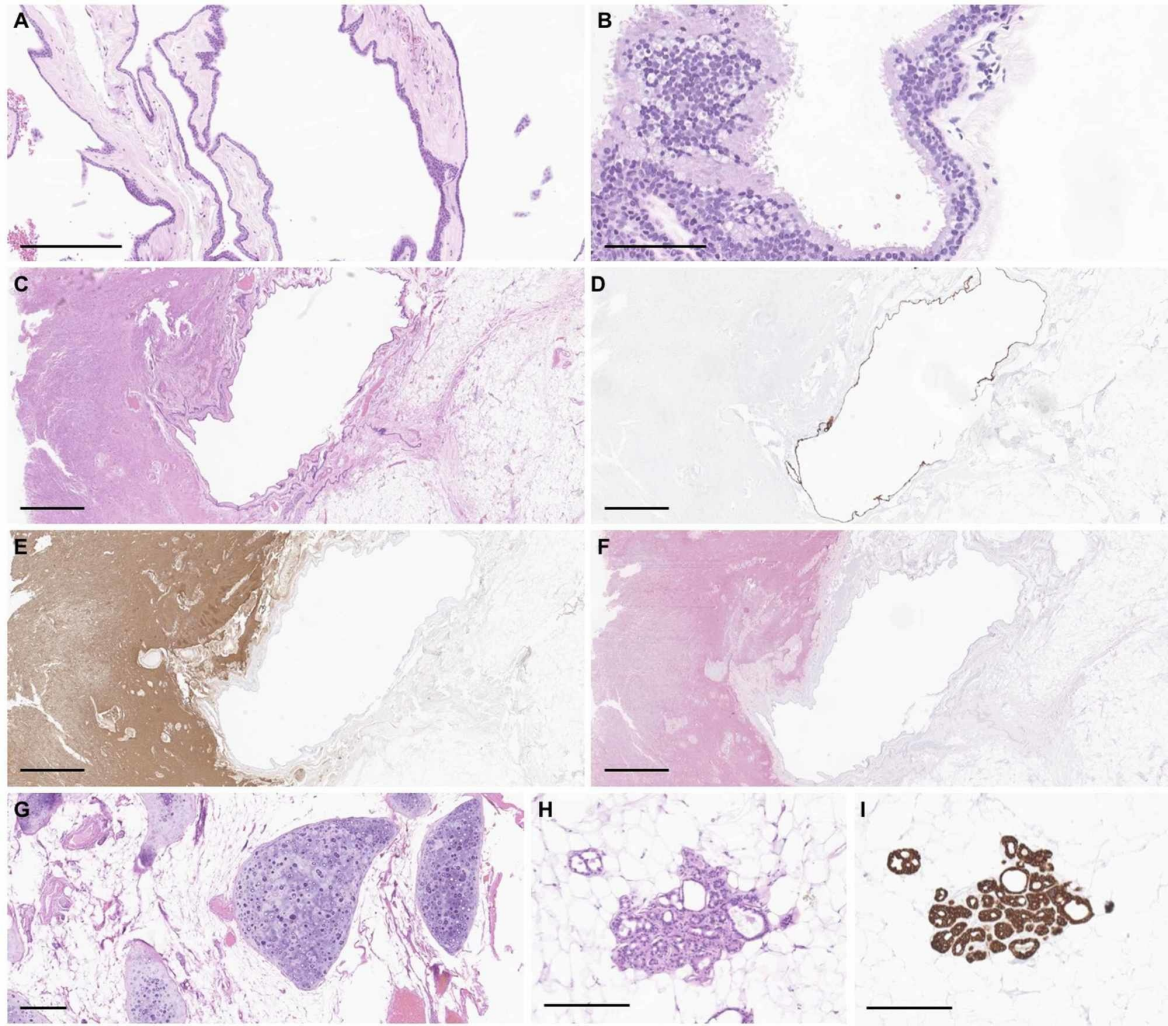
Pathology Rostral thoracic mass. (A) Squamous lined epidermal component with (B) second cystic component composed of ciliated, GI-type glandular tissue. Caudal thoracic mass. (C) Adipose tissue with squamous-lined cyst and disorganized neuropil. The cystic component is immunoreactive for cytokeratin CAM5.2 (D), and the neuropil-like region is immunoreactive for glial fibrillary acidic protein (E) and S100 (F). Other regions show immature cartilage (G) and glands (H) which are immunoreactive for cytokeratin CAM5.2 (I). Parts A, H, I ×100 total magnification, scale bars = 200 microns; part B, ×200 total magnification, scale bar = 100 microns; Parts C-F, ×20 total magnification, scale bar = 500 microns; part G, ×50 total magnification, scale bar = 200 microns.

Microscopic sections of both lesions revealed fibrous and adipose tissue with cystic spaces. The rostral lesion was composed predominantly of squamous-lined cyst with mature glial tissue, focal cartilage, GI-type glandular elements, and ciliated cystic spaces. The larger, caudal intramedullary lesion demonstrated admixed nerve, disorganized smooth muscle, immature appearing cartilage, squamous-lined cysts, and glandular elements (Figure 3). The diagnosis of both lesions was most consistent with mature teratoma. 

## Discussion

Spinal teratomas are rare, with large spinal tumor case series citing a less than 0.5% incidence among all spinal tumors [6]; however, awareness of this pathology is increasing. Indeed, a recent comprehensive review has documented approximately 150 cases reported in the adult literature [2]. Despite a wide range of radiographic presentations, with lesions extending from upper cervical [7,8] to the sacrum [9,10], and a similar wide range of clinical presentations, with patients as young as 18 years [11] and as old as 85 years having been reported [12], there is a general consensus that mature teratomas are slow-growing tumors, with overall recurrence rates after subtotal resections of approximately 6% and time to recurrence averaging 88.2 months [2]. Immature teratomas of spinal cord in the adult however are far more aggressive. Indeed, while there is no consensus regarding the best course of treatment for immature teratomas given their rarity, the only two reported cases have argued for maximal resection as the first line of treatment since adjuvant therapies remain unproven. Despite this, both reported cases demonstrated clinically significant recurrence within 10 months despite near total resections [4,5].

The presence of multiple lesions presenting in the same patient is exceedingly rare among the reported cases. Indeed, to our knowledge, this is only the second such report. Despite our initial suspicion that the presence of multiple simultaneous lesions would correlate with an immature phenotype, neither the previously reported multiple lesion patient [3] nor ours had either immature or malignant features. Similarly, neither previously reported immature teratoma case initially presented with more than a single lesion, though one patient recurred with two lesions [5]. 

The current consensus for the treatment of mature spinal teratomas is maximal safe resection; however, in the case of single solitary lesions, there is a treatment bias in that the diagnosis itself requires surgical intervention. It is not as clear that this is the optimal course of action in the setting of multiple lesions, particularly when not all lesions are symptomatic. With the presence of multiple lesions and an intraoperative frozen section diagnosis of teratoma, the concern is that the presence of multiple lesions suggests an immature pathology, which necessitates a more aggressive approach. However, as seen in our patient, this is not necessarily the case. Indeed, even with multiple spinal teratomas, it is still likely that all of them are mature. Thus, the risk of developing a new neurologic deficit from a complication after operating on an asymptomatic level, as well as the low likelihood of progression if an asymptomatic level is left untouched, could favor a more conservative approach. However, given the still relatively small numbers of both reported immature spinal teratomas and spinal teratomas presenting with multiple lesions, we would argue that a suspicion for an immature pathology should still be kept for patients with multiple lesions. 

In our patient, we felt we could safely resect the rostral asymptomatic lesion and thus give him the highest chance for a cure regardless of whether the final pathology was suggestive of either a mature or immature teratoma. However, given his postoperative complication, it is debatable whether a better approach would have been to minimize the operative time by avoiding this level unless pathology from the caudal symptomatic lesion was suggestive of an immature teratoma. In the very least, we feel that this would have been a reasonable approach. 

## Conclusions

Our case suggests that while rare, multiple mature teratomas of the spine can occur and, indeed, can occur in the adult. As such, it should be included in the differential of multiple spinal lesions and planned for accordingly, with particular attention being paid to the fact that multiple lesions have not thus far demonstrated a higher risk of an immature pathology. While maximal safe resection remains the goal, in cases of multiple mature spinal lesions, particularly when not all are symptomatic, this must be weighed against the risk of worsened neurologic deficit and the slow rate of growth. 
